# Maladie de kaposi classique avec atteinte surrénalienne: un nouveau cas

**DOI:** 10.11604/pamj.2014.17.234.3901

**Published:** 2014-03-27

**Authors:** Hanae Bouzidi, Salim Gallouj, Sanae Krich, Fatima Zahra Mernissi

**Affiliations:** 1 Service de Dermatologie Vénérologie, CHU Hassan II, Fès, Maroc

**Keywords:** Maladie de kaposi, VIH, atteinte surrénalienne, Kaposi disease, HIV, adrenal involvement

## Abstract

La maladie de kaposi est une pathologie connue généralement dans sa forme épidémique associée au sida ou celle endémique présente dans l'Afrique subsaharienne. La forme classique de cette maladie ou dite également méditerranéenne n'est pas bien connue étant assez rare. Elle touche les hommes âgés HIV séronégatifs originaires de l'Europe centrale, l'Europe de l'Est et les méditerranéens. Elle se manifeste essentiellement par une atteinte cutanée, éventuellement muqueuse mais l'atteinte viscérale reste moins fréquente, dominée par l'atteinte osseuse, pulmonaire et gastrique. La localisation surrénalienne est inhabituelle et très rare. Nous rapportons ce nouveau cas pour mettre le point sur cette pathologie rare et signaler cette localisation atypique.

## Introduction

La maladie de kaposi (MK), est une maladie proliférative chronique à double composante vasculaire et fibroblastique induite par le 8ème virus de l'herpès humain (HHV-8). Quatre variantes de la MK sont décrites, dont la MK classique ou dite également méditerranéenne qui touche les hommes âgés HIV séronégatifs originaires de l'Europe centrale, l'Europe de l'Est et les méditerranéens. Elle se manifeste essentiellement par une atteinte cutanée sous forme de papulo-nodules angiomateux sur un terrain de lymphoedème au niveau des extrémités. Elle est considérée la plus indolente avec une évolution généralement lente. L'atteinte viscérale est peu fréquente dominée par l'atteinte osseuse sous-jacente aux lésions cutanées, l'atteinte pulmonaire puis gastrique. La localisation surrénalienne est inhabituelle et très rare. Nous rapportons un cas particulier d'une MK classique avec une atteinte de la glande surrénalienne.

## Patient et observation

**Figure 1:**
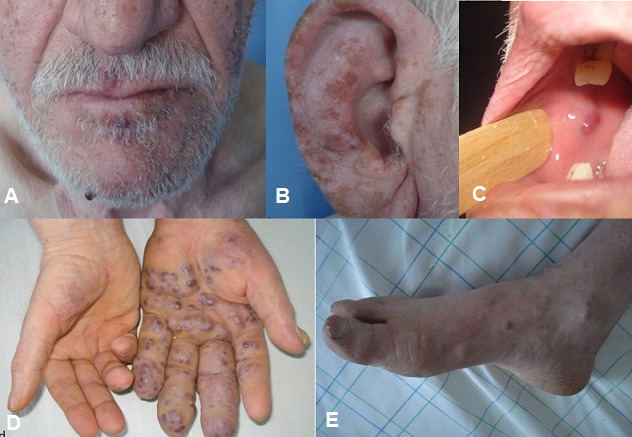
A, B: nodules angiomateux au niveau de la face; C): de la muqueuse buccale; D,E): des extrémités distales

**Figure 2:**
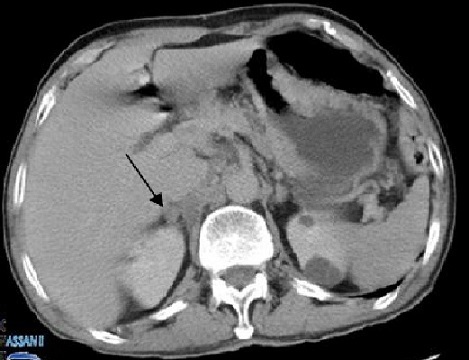
Nodule tissulaire surrénalien droit de 2,6cm

Monsieur F M âgé de 70 ans, originaire du Maroc, sans antécédents pathologiques notables, consultait pour des nodules angiomateux de la face, des extrémités et de la muqueuse buccale avec un oedème de la main gauche (Figure 1) évoluant dans un contexte de conservation de l’état général. La biopsie cutanée avec étude immunohistochimique était en faveur d'une maladie de kaposi. La sérologie HIV était négative. La fibroscopie oeso-gastro-duodénale avait montré la présence de 4 lésions mesurant chacune 10mm au niveau gastrique avec une biopsie en faveur d'une MK. La tomodensitométrie thoraco-abdominopelvienne avait objectivé un nodule surrénalien droit de 26mm (Figure 2). Le patient a été classé comme forme méditerranéenne de la MK avec atteinte multiviscérale, mis sous polychimiothérapie à base d'Adriablastine, Bléomycine et Vinblastine. Après 6 cures on a eu une amélioration clinique, endoscopique et une stabilité radiologique sans l'apparition de nouvelles lésions avec un recul de 2 ans.

## Discussion

La MK est une maladie proliférative multifocale, d'expression cutanée et viscérale impliquant diverses cellules mésenchymateuses [1, 2]. Elle est classée en 4 formes épidémiologiques: 1) MK classique, touchant le plus souvent les hommes âgés originaires de l'Europe centrale, l'Europe de l'Est et les méditerranéens avec une prédominance chez la population juive; 2) MK endémique (Afrique subsaharienne); 3) MK iatrogénique associée à une immunosuppression; 4) MK épidémique (associée au SIDA/ HIV) [3]. La MK classique se présente habituellement sous forme de macules violines et érythémateuses qui évoluent lentement vers des plaques et des nodules angiomateux. Ces lésions sont habituellement localisées au niveau des extrémités distales [1]. La MK endémique atteint une population plus jeune avec une évolution rapide, localement agressive et s'accompagne souvent d'une extension aux ganglions lymphatiques et d'une atteinte viscérale. La maladie est alors assez souvent mortelle [4]. la MK Iatrogénique survient chez des sujets soumis à des traitements immunosuppresseurs au long cours, dans le cadre ou non de transplantation d′organes, se manifeste par plusieurs lésions cutanées sur les extrémités distales et suit une évolution bénigne [5, 6]. La MK associée au SIDA/HIV se présente sous des formes cutanées et muqueuses extensives avec atteinte ganglionnaire et viscérale et peut conduire rapidement au décès [7]. La localisation viscérale est surtout l'apanage des formes endémique et épidémique de la MK. Dans la MK classique, elle est plutôt rare, dominée par l'atteinte osseuse reconnue de longue date, estimée à peu près d'un tiers des cas [8] prédominent aux extrémités et en regard des lésions cutanées surtout lorsque le plan osseux est superficiel [9]. L'atteinte digestive est également importante, d'où la nécessité de rechercher le sang dans les selles par le test de Weber [10], Le taux le plus important d'atteinte gastro-intestinale a été rapporté dans une série grecque chez 81,6% des patients [11]. L'atteinte pulmonaire parenchymateuse et pleurale moins fréquente a été rapportée dans quelques séries [12, 13]. Les autres organes du corps ne semblent pas à l'abri du développement de lésions Kaposiennes [14, 15], mais très peu de cas sont rapportés dans la littérature notamment pour la localisation surrénalienne, seulement 2 cas dont une au cours de la MKC et l'autre au cours de la MKC avec HIV [16]. Notre observation constitue un nouveau cas. Le patient était asymptomatique sur le plan clinique et pourtant le bilan lésionnel avait objectivé une atteinte multifocale nécessitant une thérapie lourde a base de chimiothérapie et un suivi régulier. En effet le caractère néoplasique de la MK classique a été longtemps discuté sans pour autant répondre à la question: s'agit il d'un sarcome ou d'une hyperplasie réactionnelle ‘ La MK était jusqu’à récemment intitulée sarcome de kaposi et cette dénomination lui est restée en anglais. Un sarcome est une tumeur dérivée du mésoderme et qui émerge dans les tissus et le mésothélium des viscères. Des données nouvelles sur la MK ont largement remis en cause sa classification au sein des sarcomes: la MK ne serait pas un cancer typique métastasant à partir d'une tumeur clonale localisée, mais une prolifération soit indolente, soit agressive, mais à caractère multicentrique. Elle est susceptible de régresser totalement et spontanément au cours de la MK classique avec un mode d’évolution très lent et une très faible mortalité [17, 18], raisons pour lesquelles le terme de maladie de kaposi et préférable à celui de sarcome de kaposi. Mais La présence des atteintes extra-dermatologiques bien quelles soient rares dans la MK classique, incite à pousser les explorations même en l'absence de signes d'appel. La survenue d'une atteinte viscérale parait corrélée à l'existence de lésions muqueuses [1, 5]. Ces résultats méritent d’être confirmés par des études multicentriques à fin d’évaluer l'incidence réelle de la MKC et de préciser ses caractéristiques épidémio-cliniques. Des études prospectives seraient également d'un grand intérêt pour mieux évaluer les protocoles thérapeutiques et le profil évolutif de la maladie.

## Conclusion

L'atteinte surrénalienne dans le cadre de la MK classique est très rare, bien qu'elle soit asymptomatique, son exploration est importante dans la classification de la maladie et sa prise en charge thérapeutique.
